# Marine Neurotoxins’ Effects on Environmental and Human Health: An OMICS Overview

**DOI:** 10.3390/md20010018

**Published:** 2021-12-23

**Authors:** Sophie Guillotin, Nicolas Delcourt

**Affiliations:** 1 Centre Antipoison et de Toxicovigilance, Pôle de Médecine d’Urgences, Centre Hospitalier Universitaire de Toulouse, 31059 Toulouse, France; guillotin.s@chu-toulouse.fr; 2 INSERM UMR 1295, Centre d’Epidémiologie et de Recherche en Santé des POPulations, Faculté de Médecine, 37 Allées J. Guesde, 31000 Toulouse, France; 3 INSERM UMR 1214, Toulouse NeuroImaging Center, Place du Dr. Joseph Baylac, 31024 Toulouse, France

**Keywords:** marine neurotoxins, HAB, genomics, transcriptomics, proteomics, metabolomics, human toxicology, food safety, ecotoxicology

## Abstract

Harmful algal blooms (HAB), and the consequent release of toxic metabolites, can be responsible for seafood poisoning outbreaks. Marine wildlife can accumulate these toxins throughout the food chain, which presents a threat to consumers’ health. Some of these toxins, such as saxitoxin (STX), domoic acid (DA), ciguatoxin (CTX), brevetoxin (BTX), tetrodotoxin (TTX), and β-N-methylamino-L-alanine (BMAA), cause severe neurological symptoms in humans. Considerable information is missing, however, notably the consequences of toxin exposures on changes in gene expression, protein profile, and metabolic pathways. This information could lead to understanding the consequence of marine neurotoxin exposure in aquatic organisms and humans. Nevertheless, recent contributions to the knowledge of neurotoxins arise from OMICS-based research, such as genomics, transcriptomics, proteomics, and metabolomics. This review presents a comprehensive overview of the most recent research and of the available solutions to explore OMICS datasets in order to identify new features in terms of ecotoxicology, food safety, and human health. In addition, future perspectives in OMICS studies are discussed.

## 1. Introduction

Marine biotoxins are phycotoxins, toxic substances produced mostly by certain species of toxigenic microalgae (also called toxigenic phytoplankton), or toxins produced by bacteria (cyanobacteria, exogenous, or endosymbiotic bacteria). These biotoxins accumulated by primary producers are integrated into the marine food chain. They can be present in various aquatic organisms, such as bivalve shellfishes (filters: mussels, oysters; burrowings: scallops, cockles, clams, ark shells; filters and burrowings: razor clams, etc.), fishes, or other marine organisms (gastropods, marine mammals). Marine biotoxins pose a serious threat to human health, as exposure to these marine biotoxins, mostly from ingestion of contaminated seafood, can possibly cause symptoms based on the type of biotoxins. Diarrhetic shellfish poisoning (DSP), paralytic shellfish poisoning (PSP), amnesic shellfish poisoning (ASP), neurotoxic shellfish poisoning (NSP), and ciguatera fish poisoning (CFP) are the most reported [1,2]. In mammals, acute exposure to marine biotoxins mainly causes digestive symptoms, but several types of marine biotoxins can also cause acute and chronic neurological signs. These toxins include saxitoxin (STX), domoic acid (DA), ciguatoxin (CTX), brevetoxin (BTX), tetrodotoxin (TTX), pinnatoxin (PnTX) groups, and β-N-methylamino-L-alanine (BMAA) [1,3,4,5].

Initially, marine biotoxins were interesting as marine drugs, for their neurological properties. Currently, due to their impact on human health, as well as the environmental and economic consequences that the proliferation of toxic algae generates, marine toxins are being widely studied by the scientific community. These studies focus on the molecular and cellular mechanisms underlying their toxicities in in vitro and in vivo models, whether cellular or animal models. These studies can help lead to understanding marine neurotoxin toxicology in humans. The bioaccumulation of these neurotoxins in the food chain is also an interesting topic. In this context, using OMICS technologies, i.e., transcriptomics, proteomics, and metabolomics, allows global information to be generated on toxin-induced molecular and cellular perturbations in cells and tissues, which are associated with adverse outcomes.

**Figure 1:**
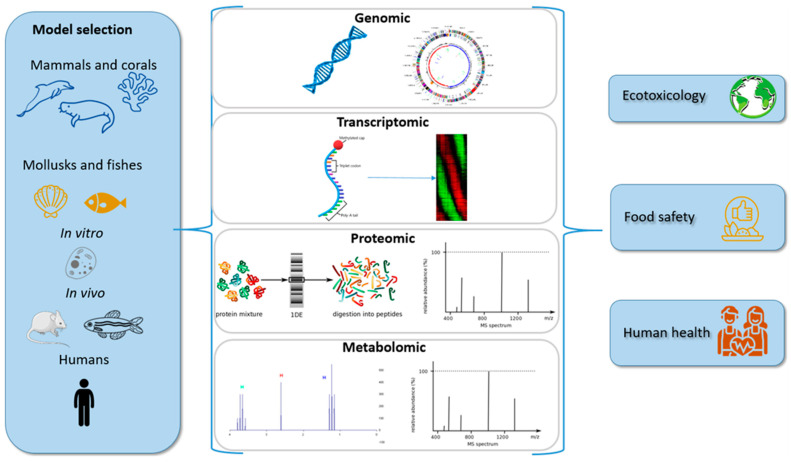
OMICS approaches in marine neurotoxin research.

In this review, we describe the different OMICS technologies available and their contribution to better characterizing the consequences of marine neurotoxin exposure in the fields of ecotoxicology, food safety, and human health (Figure 1).

## 2. OMICS Overview

“OMICS” refers to high-throughput analyses of genes, proteins, or metabolites in a biological system, and it represents an emerging field for marine biotoxin research. We found 49 OMICS research articles in regard to marine neurotoxin research, half of which were published in the last five years. Notably, we excluded a review of OMICS-based studies of marine toxins that do not affect the nervous system (notably DSP; for a review, refer to [6]), as well as reviews that study biosynthesis pathways [7].

### 2.1. Transcriptomics

Transcriptomics is the study of the complete RNA content of an organism. The information present in DNA is expressed through transcription, which may help to identify pathways associated with adverse outcomes induced by neurotoxin exposure. The contemporary techniques used to study transcriptomes are microarrays and RNA-sequencing. Microarray is a tool to measure the expression profile of multiple genes simultaneously [8]. Due to its intrinsic limitations, such as low resolution, a low dynamic quantification range, or an inability to distinguish different isoforms, this method was replaced by high-throughput next-generation RNA-sequencing technology (RNA-Seq) [9]. RNA-Seq is currently more expensive than microarrays, but it has the advantage of detecting nonannotated sequences and alternative splice variants in addition to expression levels. This method reveals the presence and quantity of transcripts in the RNA extract, and it has the potential to identify particular genes that are active at a given moment in time. Concerning marine toxin research, transcriptomics has notably been used to describe the biosynthesis pathways in aquatic producers, a research field that was recently reviewed [6,7]. Moreover, transcriptomic approaches have been applied in the field of food safety, particularly in bivalves experimentally exposed to STX and DA, and in the Japanese puffer fish *Takifugu rubripes* contaminated by TTX (Table 1).

### 2.2. Proteomics

Proteomics aims to profile the complete protein content of a biological sample, including protein modification and interaction [38]. The mainstream proteomic approach is based on mass spectrometry (MS) technology. In shotgun proteomics, the workflow is based on protein extraction from the biological sample, followed by digestion by an endoprotease (commonly trypsin, which cleaves after each lysine and arginine residue), chromatographic separation, and MS analysis. MS determines the mass-to-charge ratio (m/z) of each tryptic peptide in the complex mixture of thousands of peptides and further fragments of each peptide to allow the determination of the amino acid sequence [38]. All of these data are then computationally analyzed against protein databases that provide the identity of each protein. The latest technological advances in MS have made it possible to identify several thousands of proteins present in biological samples or in organisms [39].

To bring the necessary quantitative dimension to the characterization of protein changes, several approaches have been described. Among them, 2-dimensional electrophoresis (2DE) prior to MS is a common methodology used to separate proteins by mass and pI that can detect differentially expressed protein spots using the difference in gel electrophoresis (DIGE) method [40]. This approach was notably used to study proteome changes in murine neuroblastoma cells, in California sea lion *Zalophus californianus,* and in marine medaka *Oryzias melastigma* exposed to STX, DA, and BTX, respectively [41,42,43]. Shotgun proteomics is another approach of choice, as it leads to the quantification of thousands of proteins without a priori. The quantitation method can be based on protein labeling or label-free methods. Firstly, sample preparation can be combined with chemical labeling using isobaric tag techniques, such as isobaric tags for relative and absolute quantification (iTRAQ). This method is based on the covalent labeling of the N-terminus and side chain amine of tryptic peptides with tags of different mass. The samples are then pooled prior to fractionation and MS/MS analysis. The generated data are used to identify labeled peptides and to relatively quantify the peptides and their related proteins from the samples they originated [44]. Using this quantitative method, Reynolds et al. [45] described a dysregulation of the proteins involved in redox homeostasis, energy metabolism and ROS production after the exposure of mustard hill coral *Porites astreoides* to *Karenia brevis* and BTX. Another famous strategy was developed by Mann’s group, based on the labeling of proteins into the biological sample using stable isotopes, such as arginine or lysine ^13^C and/or ^15^N [46]. This method was chosen by Beri et al. [47] to investigate the molecular pathways perturbed by in vitro exposure of BMAA to a mouse motor neuron cell line (NSC-34). More recently, TMT labeling, an approach that allows up to tenfold multiplexing and, at the same time, isotope labeling-based quantification, was also used to study protein regulation in mouse cells exposed to STX [48]. Associated with advances in MS and bioinformatics tools, label-free quantitative proteomic approaches were developed and are now considered to be reliable, efficient methods of studying protein level changes in complex mixtures. These approaches are based on the measurement either of the MS/MS sampling rate of a particular peptide (generally the most intense) or of its MS chromatographic peak area. These values are then directly related to peptide abundance. Notably, these approaches are compatible with high-throughput quantitative analysis, but they have a lack of reproducibility, particularly when samples have a high dynamic range [49]. In the field of marine neurotoxin research, label-free quantitative proteomics has been used to monitor proteome changes in the cerebrospinal fluid (CSF) of *Z. californianus* contaminated by STX, providing a relationship between dysregulated proteins and neurodegenerative diseases (Table 2) [48].

### 2.3. Metabolomics

Finally, metabolomics is commonly used to decipher the variation in metabolites, which represent the final products of interactions between gene expression, protein function, and cellular environment [58]. In contrast to nucleic acids or proteins, metabolites encompass a set of chemically highly heterogeneous molecules [59]. The metabolites that compose the metabolome have a molecular weight ranging from 50 to 1500 kDa, and include molecules such as lipids, sugars, amino acids, nucleic acids, or steroids. Metabolome measurement relies either on nuclear magnetic resonance (NMR)-based detection or on gas or liquid chromatography (GC or LC) coupled with MS [60,61]. In actuality, NMR and MS instruments are capable of separating, detecting, and characterizing hundreds to thousands of chemicals in complex chemical mixtures, such as biofluids or tissue extracts [62]. We found four studies that used NMR and three that used MS to study the impact of marine neurotoxins (BTX and BMAA) on the metabolome changes in various toxicological models such as *O. melastigma*, zebrafish *Danio rerio*, mouse neuronal cell lines, and rats [63,64,65,66]. Interestingly, these results showed that BTX exposure led to a dysregulation of metabolites involved in neuronal excitotoxicity, whereas BMAA seemed to alter energetic metabolic pathways (Table 3). In addition, specific methods exist for system-level analysis of protein glycosylation (glycomics) and lipids (lipidomics), although these have not been used in neurotoxin research [67]. 

## 3. Paralytic Shellfish Poisoning (PSP)

PSP is one of the most studied marine intoxications and can induce neuromuscular symptoms such as muscular paralysis, myalgia, and respiratory difficulty. In very severe intoxications, muscular paralysis and dyspnea may evolve into respiratory arrest and death. About 2000 cases of PSP are recorded annually with a human mortality rate of 15%. This poisoning is due to the ingestion of seafood products containing STX, a family of nonterpene alkaloids also known as paralytic shellfish toxins (PST) [70]. STX is largely produced by marine microalgae of the genus *Alexandrium* [1]. These neurotoxins act by inhibiting the generation of action potentials in the membranes of neurons and muscle cells as STX blocks Na^+^ influx through the voltage-gated sodium channel (NaV) by binding to site 1 of the α subunit [1]. These toxins have a worldwide distribution, with reports of PSP in coastal regions of temperate and tropical areas [1]. Due to climate change, STX has become a worldwide environmental and health problem, attracting global concern [1,71].

### 3.1. Food Safety

STX may induce declined reproduction and growth rates, especially in marine bivalves, and could be a major risk of mortality in the Chilean mussel *Mytilus chilensis* [72,73]. Therefore, harmful algae bloom (HAB) has led to massive deaths and, hence, major environmental, economic, and social issues. Since mollusks are natural filter feeders on algae, they can accumulate toxins [74,75]. Although bivalves can tolerate much higher concentrations of STX than humans and other mammals, STX accumulation causes changes in the behaviors and metabolism processes of bivalves [76,77]. First, STX exposure creates huge changes in cellular homeostasis. Roncalli et al. [10] used RNA-Seq in the calanoid copepod *Calanus finmarchicus* to demonstrate a downregulation of transcripts involved in lipid biosynthesis, growth, and reproduction. These results suggested that bivalves exposed to STX have less energy available, contributing to lower egg production and egg quality. Second, the exposure led the species to protect themselves against the toxins. Several studies have shown an activation of an innate immune response after STX exposure. Detree et al. [11] used RNA-seq and quantitative reverse transcription PCR (RT-qPCR) approaches to evaluate the transcriptomic response of *M. chilensis* to STX. It appeared that pattern recognition receptors (PRRs) involved in mussel immunity, such as Toll-like receptors, tumor necrosis factor receptors, and scavenger-like receptors, were found to be strongly upregulated after STX injection [11]. The impact of STX exposure on the immune response has been confirmed in hemocytes of *M. chilensis* with qPCR analyses by Astuya et al. [12] and in the Mediterranean mussel *Mytilus galloprovincialis* using RNA-Seq by Gerdol et al. [13]. Third, STX exposure is known to induce oxidative stress in bivalve strains through the overproduction of reactive oxygen species (ROS). The antioxidant metabolism is crucial to preventing cellular oxidative damage in bivalves exposed to toxic dinoflagellates, such as *Alexandrium* sp. Two kinds of studies have described this mechanism. In a mollusk flesh study, Astuya et al. [12] evaluated the response of hemocytes from *M. chilensis* to STX exposure. The authors showed a significant increase in superoxide dismutase (SOD) and catalases transcripts in *M. chilensis* [12]. Astuya et al. [12] stated that this increase could be identified as an early biomarker of pollution. However, a year later, a predominant downregulation of the antioxidant enzymes was observed in *C. finmarchicus* [10]. Due to this ambiguity, it will be interesting to analyze the regulation of these enzymes in specific organs. Indeed, the hepatopancreas is known to be the main organ for STX uptake, whereas toxins are metabolized in the kidneys into more toxic analogs [78]. In Farrer’s scallop *Chlamys farreri* and Yesso scallop *Patinopecten yessoensis*, gene expression of glutathione peroxidases (GPx) has been specifically studied in response to STX stimulation. In the hepatopancreas of these species, STX exposure did not induce significant dysregulation of GPx, whereas most GPx were found to be upregulated in kidneys [14]. This suggests a function for GPx in protecting the kidneys against oxidative stress. In this study, the authors revealed a predominant upregulation of SODs in the hepatopancreas and the kidneys, with SOD6 as the only member being upregulated in both organs. [15]. Fourth, 131 transcripts of solute carriers (SLCs) were found to be upregulated in the hepatopancreas of *P. yessoensis* [16]. These mRNAs are translated in membrane proteins that transport many endogenous and exogenous substances such as xenobiotic toxins [79]. Xun et al. [16] stated that there could be a close relationship between the expression of SLCs and the accumulation of STX in the scallop hepatopancreas. This hypothesis could be used to develop inhibitors against the SLCs to prevent the primary effects of STX.

### 3.2. In Vitro Studies

In order to investigate the potential risk of exposure to low-dose STX in mammals, Chen et al. [41] analyzed proteome changes in murine neuroblastoma cells. The authors studied the effects of low doses of STX during 24 h of exposure (1 nM and 10 nM) using 2D DIGE and MALDI-TOF-MS analysis. Chen et al. [41] demonstrated an alteration of nine proteins, including 14-3-3 beta, alpha enolase, and cofilin 2, which are known to play a role in cell apoptotic pathways, cell skeleton maintenance, membrane potentials, and mitochondrial functions. These results are in accordance with the reports on genotoxicity and neurotoxicity induced by STX. Interestingly, the downregulation of voltage-dependent, anion-selective channel protein 1 (VDAC1) suggests a potential intracellular mechanism by which low-dose exposure to STX could modify membrane cell depolarization [41]. 

### 3.3. In Vivo Studies

Recently, Sun et al. [48] carried out a hippocampal proteomic analysis to study the impact of long-term STX exposure. Over 3 months, four groups of C57BL/6NJ mice were treated per os with 0, 0.5, 1.5, or 4.5 µg eq. STX/kg body weight. The results were obtained using TMT labeling and LC-MS/MS analysis. The expression of 23 proteins was changed, including mostly proteins involved in the development of neurodegenerative diseases, one of which is the sphingolipid metabolism pathway-related protein Smpd3. This result is particularly interesting, as the sphingolipid metabolism pathway is closely associated with the development of Alzheimer’s disease (AD) [80]. Moreover, several mitochondrial proteins involved in AD, Parkinson’s disease (PD), and Huntington’s disease, such as ATP synthase subunit epsilon and cytochrome c oxidase, were dysregulated in these conditions. Further investigations are needed to verify this molecular mechanism in STX-induced nerve damage.

## 4. Amnesic Shellfish Poisoning (ASP) 

ASP is another important neurological poisoning caused by the production of DA, a member of the kainoid group. *Pseudo-nitzschia* diatoms are the most common producers of DA [81]. In 2015, these species were found to be the most dominant algal species detected in HAB [82] and are even widely found around the world, with several strains in the Mediterranean Sea [1]. DA exerts its toxicity by activating AMPA and kainate ionotropic glutamate receptors in the central nervous system (CNS) [1]. Acute exposure to DA can cause amnesia, seizures, comas, and death in extreme cases. Chronic exposure can lead to kidney damage (mice) and impairment of fetal development in animal models, such as sea lions, monkeys, and rodents, and even cognitive deficit in humans [83]. DA is also reported as harmful for seabirds and marine vertebrates, such as *Z. californianus,* which suffer from spatial memory impairment [83,84,85].

### 4.1. Ecotoxicology

More and more animals are being found stranded on beaches. There has been an increase in interest from the general population on this subject, particularly in California with *Z. californianus.* Different labs have been looking for biomarkers to understand these events and try to prevent them. Mancia et al. [19] analyzed RNA from the blood samples of 73 sea lions, which they distributed into four groups: DA toxicosis (DAT), leptospirosis infection, healthy, and outgroup. Three genes of interest were identified in the DAT group: *presenil-2*, *β-site APP-cleaving enzyme 2,* and *TNFAIP6*, all dysregulated compared to the leptospirosis infection group. The first two genes are remarkably interesting because the genes are also detected in AD studies [86]. *TNFAIP6* is known to be principally regulated by tumor necrosis factor α and interleukin-1 [87]. Due to its significant sensitivity, *TNFAIP6* could be considered a biomarker. In another work, Neely et al. [42] refined these results by comparing the plasma proteomic profiles of chronic DAT and non-DAT populations using 2D DIGE and LC-MS/MS. Eleven downregulated proteins were highlighted related to a charge-form of apolipoprotein E (ApoE). The link between ApoE and AD is often being studied at present, especially the subtype ApoE ε4. Indeed, patients carrying this allele showed a substantially higher risk for AD, with a lower age of clinical onset [88]. Neely et al. [42] stated that as a neurodegenerative disease, chronic DAT progression could be explained by changes in ApoE charge form distribution. Using an independent test set, ApoE was qualified as a biomarker with an excellent sensitivity but a low specificity [42]. However, coupled with eosinophil counts, which were found to be increased in DAT, the authors were able to discriminate the DAT population from the non-DAT population [89]. Later, the same team also studied CSF proteins using a pilot label-free LC-MS/MS analysis. Six proteins of interest were demonstrated to be upregulated: complement C3, complement factor B, dickkopf-3, neuron cell adhesion molecule 1, gelsolin, and neuronal cell adhesion molecule (NCAM) [50]. The first two proteins are complement proteins, already studied as candidate biomarkers for several major neurodegenerative diseases [90] and thus potential biomarkers to describe chronic changes. The other four proteins are known to be implicated in neuroprotection, neurogenesis, and synaptogenesis. Thus, their increase may be a reflection of neuroregeneration following an excitotoxic insult induced by DA.

### 4.2. Food Safety

Toxin accumulation in shellfish has adverse economic impacts, leading to harvest closures caused by the human health problems. Herein, we focus on three transcriptomic studies of ASP indicators in shellfishes. First, the exposure on the digestive gland of *M. galloprovincialis* to the DA-producing *Pseudo-nitzschia* has been studied by RNA-Seq [20]. Most DA accumulates in the mussel’s digestive gland since it is the main site of xenobiotics detoxification [91,92]. Pazos et al. [20] observed an upregulation of genes involved in detoxification processes, in the response against oxidative stress (e.g., sulfotransferases, glutathione synthesis) and in immunological processes (*C1q domain*, *fibrinogen C-terminal globular domain*). Second, Ventoso et al. [21] observed most of these dysregulations on the digestive gland in the queen scallop *Aequipecten opercularis* using RNA-Seq. Indeed, there was an upregulation of genes involved in immunological and detoxification processes. However, the SLC6 family was an exception, with 48 downregulated genes. Whether it is for mussels, diatoms, or zebrafish models, an upregulation of the SLC6 family has been observed in ASP exposure or production [17,20,93]. Third, Ventoso et al. [22] confirmed this trend of an upregulation of proteins involved in detoxification and immunological processes in the king scallop *Pecten maximus* using the same approach.

### 4.3. In Vivo Studies

We focus on two studies using the same model, *D. rerio* [17,94]. These studies reported a basis for identifying pathways of DA-induced injury as well as biomarkers of several DA exposure levels. The studies identified transcripts related to apoptosis and neurological pathways. Indeed, Lefebvre et al. [17] stated a neuronal apoptosis on the zebrafish brain after acute exposure to DA at symptomatic and asymptomatic doses by microarray and RT-qPCR analyses. Most dysregulated genes are those involved in transcriptional regulation, because DA exposure activates glutamate receptors in the CNS. Among these genes, Lefebvre et al. [17] showed an upregulation of *c-Fos*, *c-Jun,* and *C/EBP*, transcription factors that may affect memory formation. These factors are hallmarks of DA-induced neurobehavioral excitotoxicity [1]. Moreover, one gene was markedly downregulated in the low dose exposure, *ndrg4*. As a member of the N-myc downstream regulated gene (NDRG) family, its specific function remains unclear, but its expression has been shown to be dramatically decreased in the AD brain compared with normal brains in human studies [18]. It would be interesting as a biomarker because of clinical similarities between AD and ASP. In the second study, instead of varying the intensity of doses, Hiolski et al. [94] assessed transcriptomic profiles, using microarray and RT-PCR approaches, for 36 weeks with low-level repetitive DA exposure. The authors observed a similar profile of dysregulations to that found by Lefebvre et al. [17]. Indeed, five genes of interest, *gria2a*, *nrxn2a*, *appa*, *nfkbiab,* and *bcl2L1*, have emerged. *Gria2a*, encoding for the AMPA glutamate receptor, was downregulated at the 2-week time point, explaining the short-term compensatory response to elevated glutamatergic activity. *Nrxn2a* was also shown to be upregulated at the 2-week time point. Linked to AD, dysregulation at this time suggested an alteration of synaptic signaling and/or neural disruption [95]. Finally, the last three genes are involved in neuroprotective, anti-inflammatory, and apoptotic responses.

## 5. Neurotoxic Shellfish Poisoning (NSP)

NSP is caused by BTX, toxins principally produced by dinoflagellates such as *K. brevis* [96]. BTX are lipophilic, cyclic polyether compounds that exhibit their toxic effects by binding and persistently activating subunit 5 of NaV in nerve, skeletal, and cardiac cells [1]. These neurotoxins lead to massive fish deaths, marine mammal mortalities, and contaminated shellfisheries [1]. After human consumption, it results in neurological troubles (oral paresthesia, dysarthria, vertigo, ataxia, walking disorders), but hospitalization is rare [70]. Contrary to the other neurotoxins, inhaled neurotoxins have been demonstrated to be associated with respiratory irritation and pose an additional threat to asthmatics [1]. Financially, the economic impact in the USA was estimated to be at least USD 82 million a year [97].

### 5.1. Ecotoxicology

HAB have been reported for centuries along the coast of Florida [98]. These blooms may induce evolved resistance to BTX in a marine mammal across several generations. Herein, we focus on two threatened species in Florida affected by these red tides, the common bottlenose dolphin *Tursiops truncatus* and the Florida manatee *Trichechus manatus latirostris*. Genomic and transcriptomic approaches have been used to analyze how these species survive red tides. Traditionally, molecular studies have been limited to a few candidate genes. Herein, the application of sequencing has led to a drastic increase in gene candidates. Therefore, these results can aid researchers in further understanding the health effects of HAB on marine species and improve species rehabilitation practices and the treatment of HAB illnesses [23]. First of all, Cammen et al. [24] compared, by restriction site-associated DNA (RAD) sequencing, the dolphins that died during unusual mortality events due to HAB exposure with those from the same geographic areas that survived HAB events. Some genes of interest that may be evolutionary targets for BTX resistance have been identified, principally several members of the MHC family known to play a role in antigen presentation to T-cells. No biological functions have been found to be associated with BTX resistance. Cammen et al. [24] hypothesized that although BTX are unlikely to be antigenic due to their small size, these toxins may act as haptens that induce an inflammatory immune response [99]. Later, a study on white blood cells, collected on manatees rescued from a red tide, confirmed the involvement of the immune response [23]. Using RNA-Seq, Lazensky et al. [23] showed that immune response (inflammation, wounds, and injuries), cell proliferation, and apoptosis were the most predominant cellular pathways dysregulated (e.g., *OSCAR* 12 fold) by red tide. Furthermore, neurodegenerative pathways were particularly affected. Indeed, there was a downregulation of many genes, acting on cognitive functioning, neurotransmitter release, and neuronal and synapse activity (*MTMR2*, *ANK2*, etc.), of which the most highly downregulated gene was *PCLO* by a factor of 977. *PCLO* is associated with neuronal and synapse loss, neurotransmitter release, and cognitive dysfunction [100,101]. Therefore, these may serve as biomarkers of mammals exposure to HAB. Moreover, by threatening reef-building corals, HAB also exerts indirect deleterious effects on these marine mammals. Indeed, these corals already suffer from rapid degradation due to ocean acidification and elevated sea surface temperatures, these same effects potentializing the toxicity and the growth of *K. brevis* [102]. Thus, quantitative proteomics is also studied using the ITRAQ approach and LC-MS/MS analysis on the *P. astreoides* population with *K. brevis* treatment. This study showed a downregulation of redox homeostasis, protein folding, energy metabolism, and ROS production [45]. The disruption of intra- and intercellular Ca^2+^ maintenance, due to the mechanism of action of BTX, may serve as the basis for this broad proteomic response. Indeed, Reynolds et al. [45] observed a dysregulation of 3 phosphoinositide-dependent protein kinase 1, which modulates the release of sequestered calcium ions into the cytosol, and of the myosin-2 essential light chain, which functions in calcium binding. 

### 5.2. In Vivo Studies

Walsh et al. [25] reported the hypothesis of another mechanism for BTX: BTX-6 binding on the aryl hydrocarbon receptor (AhR) on the liver and brain tissues of mice. As dioxin, as a prototypical ligand of AhR, is the first step in a cascade pathway producing major changes in gene expression, Walsh et al. [25] hypothesized that BTX-6 might produce similar genomic-wide changes in expression. This hypothesis has not been demonstrated. Indeed, only 29 genes were dysregulated [25], a lower number than a classic dioxin response, where changes in the expression of hundreds of genes have been observed in cultured human cells [103]. The dysregulation of these 29 genes was more consistent with a general acute phase response to toxic agents. Walsh et al. [25] stated that BTX-6 might act as a modifier of these responses by interfering with the binding of other effector ligands. Another study used *O. melastigma* to describe dysregulations of proteins, metabolites, and neurotransmitters induced by sub-lethal doses of BTX-1 [43]. Tian et al. [43] analyzed proteins in gills and brain by 2D DIGE and MALDI-TOF/TOF, as the loss of normal gill functions led to fish deaths and as the brain is the main target of the neurotoxin [43]. Changes in cell homeostasis have been demonstrated. Indeed, there was a dysregulation of calcium homeostasis, a dysregulation of myosin-like proteins involved in calcium uptake and transduction, and a decrease in calreticulin, a modulator in the endoplasmic reticulum (ER) [43,104]. Therefore, all these events led to directly relating the changed expression of calcium ion-binding proteins to BTX-1 exposure. Moreover, the decrease in gelsolin and cytokeratins may illustrate cell and tissue damage induced by BTX. Finally, an interesting result has led to initiating further studies in metabolomics: an increase in glutamine synthetase, which suggests an increased level of glutamate in accordance with the excitotoxic effect of BTX. Indeed, two recent studies have been published on a metabolomic approach. First, one study focused on 43 classical neurotransmitters, amino acids, and metabolites using LC-MS/MS [63]. Yau et al. [63] demonstrated that the molecules related to the activation of the NaV, NMDA receptors, and cholinergic neurotransmission contribute to discriminating between treated and control *O. melastigma*. All the dysregulations may be explained by the activation of NaV-disturbing serotonin–norepinephrine–dopamine reuptake metabolisms in the CNS [105]. This leads to NMDA receptor activation, neuronal excitotoxicity, and also the metabolism of proteins involved in NMDA receptors [105]. Second, Annunziato et al. [64] used HRMAS-NMR analysis on zebrafish embryos after BTX-2 exposure. Similarly, the authors demonstrated neuronal excitotoxicity in response to the mechanism. In addition, BTX exposure has been interrelated with pathways of carbohydrate and energy metabolism, as well as consequent oxidative stress caused by the toxin. These observed metabolic alterations have been integrated into a model consistent with well-characterized neurotoxicity as well as with pathways that may provide aspects including respiratory and pulmonary effects and associated cellular energetics.

## 6. Ciguatera Fish Poisoning (CFP)

CFP is the most commonly reported natural marine toxin related to global illness [106]. The primary source of this poisoning is benthic dinoflagellates of the genus *Gambierdiscus* and *Fukuoya* [106]. This exposure is common in tropical countries and impacts between 50,000 and 500,000 people annually [106]. CFP is caused by CTXs. CTXs are liposoluble cyclic polyether ladder compounds, potent secondary metabolites, and NaV activators and voltage-gated potassium channels blockers. CFP results in acute symptoms including digestive troubles, muscular and joint aches, cold allodynia, arrhythmias, and, rarely, respiratory paralysis [106]. However, 20% of CFP cases may progress to chronic ciguatera, with chronic asthenia that can last for years [107]. 

### 6.1. In Vitro Studies

We focus on a transcriptomic analysis in primary neurons of mice in culture using whole mice genome microarrays, exposed to CTX3C treatment for 6 h, 24 h, or 72 h [26]. Dysregulated effects are exclusively the consequence of its action as an NaV activator, as all these effects were avoided by preincubation with TTX. No genes of interest were found to be dysregulated after 6 h treatment. After 24 h, dysregulation of the opioid receptor µ1 was observed. Interestingly, this activation is required for the perpetuation of hypothermia [108]. Since CTX-1 induces pronounced hypothermia that lasts for about 7 h [27], it is suggested that the downregulation of the opioid receptor µL was compensating for this effect. Finally, after 72 h of treatment, there was a downregulation of the gonadotropin-releasing hormone pathway. Rubiolo et al. [26] stated that it could result in the downregulation of immediate early genes, such as *Fos*, *Jun,* and *early growth response* (Egr) isoforms, as found with a CTX-1 treatment in the mouse brain [27]. It would be interesting to confirm this effect on early genes with other models.

### 6.2. In Vivo Studies

Concerning in vivo studies, a group based in South Carolina in the USA carried out different studies on mice (blood, brain, and liver) to understand acute CTX exposure [27,28,29]. Each study used a transcriptomic approach by microarray and confirmation by RT-qPCR. This group reported a dysregulation of immune (CD molecules and cytokines) and inflammation systems in acute exposure, specifically a dysregulation of interleukin 1b (Il-1b) [27,28,29]. More precisely, downregulations of the eosinophil-specific gene (*Ccr3*) and eosinophil-associated ribonucleases and an upregulation of *Ccl24* could indicate eosinophil recruitment to inflammatory sites [28]. Combined with an upregulation of the immune system driven by histamine [109], the authors stated that CTX has a similar Th2 immune response to those observed in asthma. Moreover, this group demonstrated a dysregulation of genes involved in phase II detoxification metabolism (involving enzymes such as glutathione reductase and glutathione-S-transferase) [28], but also in phase I with cytochromes 2 and 4 [29]. Different hypotheses were stated to explain the variations of cytochromes: their role in the detoxification of xenobiotics, their role in lipid metabolism—which is known to be activated in response to CTX exposure—or their dysregulated activity induced by hypothermia.

### 6.3. Human Toxicity

In a recent study on CTX exposure, the group based in South Carolina were able to study the whole blood of patients with ciguatera-induced chronic inflammatory response syndrome with the same transcriptomic approach [30]. Ryan et al. [30] again reported important dysregulations of immunological pathways. More precisely, two DQ haplotypes, *DQ2* and *DQ8*, have been found to be overrepresented in many inflammatory disorders [110]. Furthermore, Ryan et al. [30] confirmed an indirect downregulation of Il-1b due to a decrease in Toll-interacting protein, a modulator of Il-1b [111]. Thus, Il-1b might be a good indicator in both acute and chronic exposures.

## 7. β-N-Methylamino-L-alanine (BMAA)

BMAA is a nonproteinogenic amino acid, first discovered in the queen sago *Cycas circinalis* in 1967 [112]. More recently, studies have discovered BMAA in diatoms and dinoflagellates [113,114,115]. There are two potential mechanisms by which BMAA may cause neurological injuries: an excitotoxic one by acting as an agonist towards glutamate receptors and a metabolic one by misincorporating into cellular proteins [5,114]. Environmental exposure may contribute to the development of neurodegenerative disorders such as ALS, as suggested in France in the Thau Lagoon [116].

### 7.1. In Vitro Studies

The studies are focused on the two potential mechanisms. First, through a cell-*free* in vitro protein followed by a quantitative LC-MS/MS analysis, one study demonstrated that BMAA is both incorporated directly into the protein backbone during de novo synthesis and attracted to the three-dimensional structure [51]. An incorporation of other amino acids such as selenocysteine [52] and canavanine [117] has already been shown. Therefore, Glover et al. [51] stated that this misincorporation occurs when structurally similar amino acids such as serine or alanine are deficient. Nevertheless, an analysis of NSC-34 cells using the SILAC approach followed by nanoLC-MS/MS did not confirm this misincorporation [47]. Second, two molecular pathways were found to be dysregulated in several cells: excitotoxic and neurodegenerative pathways. Two teams described an excitotoxic effect with dysregulations of apoptosis/autophagy, aggregation/degradation proteins, and cell homeostasis by using a dual-OMICS approach: transcriptomic (microarray and RT-qPCR) and proteomic (nanoLC-MS) [31,53]. Moreover, this dysregulation was also observed with glutamate exposure [31]. The neurodegenerative pathway was revealed by several dysregulated proteins and transcripts such as 3-hydroxyacyl-CoA dehydrogenase type-2, ubiquilin-4, Nck-associated protein 1, and also NRF2 oxidative stress response, known to be implicated in AD and ALS [31,47,118]. Finally, BMAA was studied with a metabolomic approach coupling NMR and LC-MS analysis. In human neuroblastoma Sh-SY5Y cells, Engskog et al. [65] observed significant alterations of polar intracellular metabolites. Indeed, there were dysregulations of the alanine, aspartate, glutamate, and arginine-proline metabolisms. Aspartate and glutamate are known as major excitatory transmitters acting in the same way as BMAA on glutamatergic NMDA receptors [119]. Furthermore, Engskog et al. [65] observed that the biosynthesis of these amino acids is also connected with intermediates in the citrate cycle. All these results indicate that BMAA preferentially interferes with fundamental metabolic pathways related to neurotransmission.

### 7.2. In Vivo Studies

With regard to proteomic studies, rats and zebrafish have been used to describe molecular pathways. Karlsson et al. [54,55,56] used rat model in three studies. In the first study, the authors analyzed changes in the striatal neuropeptide system of male and female rat pups treated neonatally (postnatal days 9–10) with BMAA (40–460 mg/kg) by nanoLC-MS/MS [54]. Two effects of BMAA exposure were observed: a dose-dependent increase in neuronal pathways and a sex-dependent increase in neuropeptides. The first variation was due to an increase in VGF-derived proteins—since VGF is a developmentally regulated secretory peptide precursor expressed by neurons [120]—and to an increase in secretogranins, precursors of peptides suggested to promote neuronal differentiation [121]. Secondly, the sex-dependent increase in favor of females indicates that female neonates might have a higher susceptibility to BMAA than male neonates. The second study applied a dose of 150 and 460 mg/kg along with a MALDI imaging mass spectrometry (MALDI IMS) analysis [55]. Karlsson et al. [55] reported a dose-dependent reduction in myelin-basic protein (MBP) in the caudate putamen and the nucleus accumbens. This protein is an essential regulator of axonogenesis, for the formation and maintenance of myelin in the CNS [122]. It could impact the learning and memory impairments observed in the animals [123]. In the third study, long-term effects were demonstrated by LC-MS/MS with the higher dose (460 mg/kg): upregulation of chaperone, cytoskeletal, and intermediate filament proteins and of proteins involved in the antioxidant defense system [56]. This enrichment may be a downstream response to the BMAA-induced intracellular formation of fibrils, hallmarks of many neurodegenerative diseases. Then, Froyset et al. [124] analyzed BMAA exposure on the second model zebrafish by nanoLC-MS/MS. Excitotoxic effects were observed, contrary to Karlsson’s studies [108,109,110], with dysregulatory functions of glutamate receptor activity/recycling, ER stress, protein biosynthesis, ROS, autophagy, and neuronal cell death. Interestingly, BMAA was found to influence the endocannabinoid system by upregulation of fatty acid amide hydrolase (FAAH). This molecule has been proposed as a therapeutic target for both ALS and PD [124,125]. Finally, metabolomics studies have led to analyzing potential mechanisms on energy and amino acid metabolism. Engskog et al. [66] found that it decreased in the blood of rats using an NMR approach. As a glutamate receptor agonist, BMAA activates the glutamatergic system and, thus, it requires energy. This energy is brought by different metabolites that were observed to be dysregulated in the study: d-glucose, lactate, 3-hydroxybutyrate, acetate, and creatine. The first three metabolites are well known in energy metabolism, and creatine is vital for normal brain development because this decrease perturbs the ATP system [126]. This metabolism change was confirmed in zebrafish embryos in a second metabolomic study with an HR-MAS-NMR approach [68]. Furthermore, Roy et al. [68] also confirmed excitotoxic effects with ROS production and a decrease in protection against oxidative stress (taurine, glutamine, and glycine). Finally, a last metabolomic study with an LC-MS/MS approach studied the incorporation of BMAA into proteins. Waidyanatha et al. [69] did not confirm this result and stated that it could be due to relatively low urinary excretion and/or the small and polar nature of the expected metabolites.

## 8. Tetrodotoxin (TTX) Poisoning

The last neurotoxin reported is TTX, produced by exogenous or endosymbiotic bacteria [1,127]. There are incidences worldwide of TTX poisoning. Traditionally associated with the consumption of *T. rubripes* in Japan, it has also been found in other marine animals, including gastropods or bivalves in Australia or on European coasts (France, Greece, etc.) [1,128]. This neurotoxin binds to the pore formed by P-loop of the NaV, leading to a blockade of nerve conduction resulting in muscle paralysis [1]. This poisoning is acutely toxic in humans, as it leads to symptoms of toxicity occurring within 10–45 min: paresthesia, motor paralysis, incoordination to cardiac dysrhythmias, unconsciousness, and respiratory failure capable of causing death [1].

### 8.1. Ecotoxicology

A team in Australia examined the southern blue-ringed octopus *Hapalochlaena maculosa* to understand TTX resistance and to identify proteins associated with this neurotoxin. This octopus attacks its preys with its venom containing the neurotoxin. In a first study, proteomes of *H. maculosa* and a producer of other toxins, the southern sand octopus *Octopus kaurna*, were compared by LC-MS/MS [57]. Whitelaw et al. [57] reported disproportionately high hyaluronidase in *H. maculosa*, suggesting that the degradation of the extracellular matrix around synapses may provide greater access for TTX [129]. Therefore, Whitelaw et al. [57] stated that a certain level of hyaluronidase might be considered an indicator of TTX production. Moreover, chitinase and serine proteases were present in both species and are known to facilitate the envenomation process [130]. The second study by DNA sequencing and RNA-Seq confirmed these observations [32]. There was a dysregulation of serine proteases, with a reduced number and expression in *H. maculosa*. Further investigations could more precisely study the differences in these serine proteases and may find correlations with toxins. Finally, this study assessed the mechanism of TTX resistance. Whitelaw et al. [32] discovered several mutations in the P-loop regions of NaV, more precisely in the NaV domain 1 of *H. maculosa*. Similar mutations have been found in Japanese pufferfish and the garter snake, known to produce neurotoxin [131,132]. Therefore, this mutation could explain the TTX production by these species with no effects on the organism. It would be interesting to study this mutation in the STX case, as these neurotoxins share the same mechanism.

### 8.2. Food Safety

In a first study using RT-PCR, Matsumoto et al. [33] demonstrated the involvement of acute phase proteins (APP), including fibrinogen and hepcidin, in the accumulation of TTX in the liver of pufferfish. Indeed, Lee et al. [133] claimed that the mRNA levels of fibrinogen-like and hepcidin-like genes, which are likely to be involved in immunity, were high in the liver, which contained high concentrations of TTX. These results were confirmed by a RNA microarray approach in the same model [34]. Feroudj et al. [34] demonstrated an upregulation of interleukin-6, known to induce the expression of APP in the liver during injury [134], and complement components such as APP. A recent study on the liver of *T. rubripes* was interested in dysregulated molecular pathways 5 days after intramuscular administration of TTX, analyzed by microarray and RT-qPCR [35]. APP were not able to be observed in dysregulation; however, Matsumoto et al. [35] demonstrated an upregulation of 69 genes, including the sodium channel β-2 subunit gene, which modulates the kinetics of channel gating as well as the stabilization and location of TTX-sensitive NaV [135,136]. With this result, the upregulation of dysferlin may explain the damage to plasmatic membranes by TTX accumulation in the liver 5 days after exposure. Finally, two other studies focused on gastropod mollusks, less studied due to the lack of genomic and transcriptomic data [36,37]. For a TTX-produced, nassariid gastropod *Nassarius succinctus,* by an RNA-Seq approach, dysregulations of detoxification and the immune system with tissue factor pathway inhibitors (HSP) were observed [36]. Moreover, Zou et al. [36] found a mutation in the NaV that could also explain TTX resistance, similar to the octopus’ resistance [32]. The second study with the gray side-gilled slug *Pleurobranchaea maculata* used PCR amplification and genotyping [37]. This study suggested that differences in TTX levels are, on the contrary, likely attributable to exogenous factors, such as differences in associated bacteria, exposure, or diet. Indeed, Yildirim et al. [37] did not find genetic dysregulations between different populations. The work to date is strongly suggestive of diet as the major source of TTX, with *P*. *maculata* accumulating TTX via feeding [137], but the lack of knowledge has not led to defining the differences precisely. It could be studied in further investigations to reduce TTX exposure.

## 9. Methods

**Figure 2:**
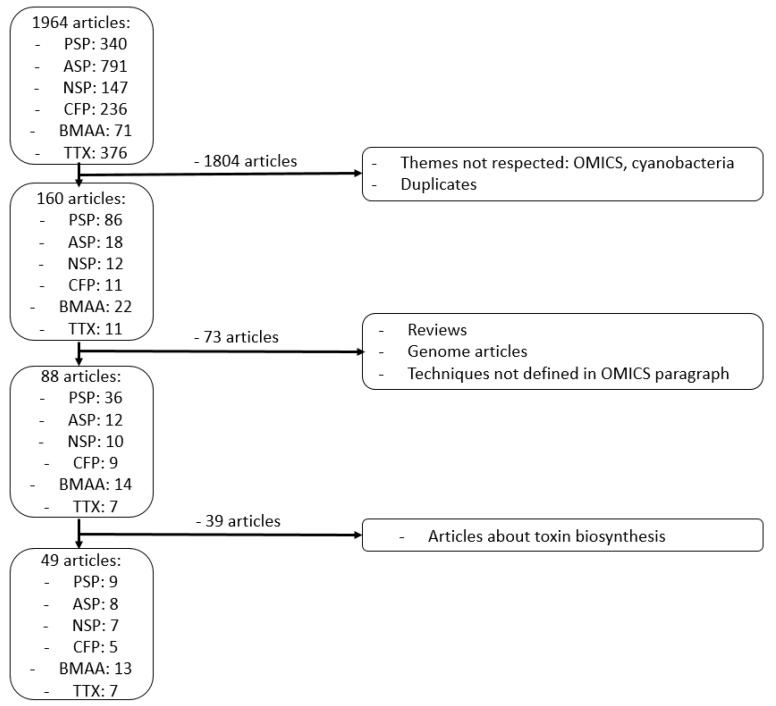
Flowchart of article selection.

To analyze the OMICS impact on the marine neurotoxins study, we searched the MEDLINE database (via PubMed) from inception to September 2021. For each neurotoxin, we associated the four approaches: “genomics”, “transcriptomics”, “proteomics”, and “metabolomics”. Moreover, for each kind of neurotoxin, several expressions were researched. For PSP, we searched “paralytic shellfish toxin”, “paralytic shellfish poisoning”, and “STX”. For ASP, we searched “amnesic shellfish poisoning”, “amnesic shellfish toxin”, and “domoic acid”. For NSP, we searched “neurotoxic shellfish poisoning”, “neurotoxic shellfish toxin”, and “brevetoxin”. For CFP, we searched “ciguatera fish poisoning”, “ciguatera fish toxin”, and “ciguatoxin”. For BMAA, we searched “BMAA” and “beta-N-methylamino-L-alanine”. For TTX, we searched “TTX” and “tetrodotoxin”. Finally, we selected articles according to different criteria (Figure 2).

## 10. Conclusions

**Figure 3:**
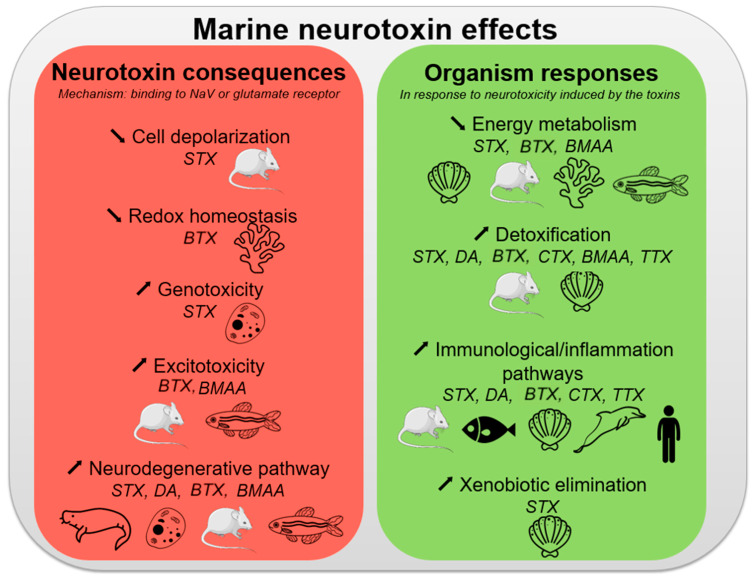
Signaling pathways involved after neurotoxin exposure. For each pathway, we associated it with the toxins and models described in the study.

The number of OMICS studies performed in the field of marine toxins research has increased substantially over the past decade. As described below, OMICS approaches have been used to generate results that provide a holistic view of an organism’s responses to the presence of toxins (Figure 3). Notably, OMICS technologies have generated new data that have led to deciphering pathways involved in toxin biosynthesis, but also to a better understanding of how these toxins exert their toxicity on their targets. However, the use of OMICS technologies in the field of marine toxin research is still in the embryonic stage and several questions have not been yet addressed using these approaches.

First, several marine neurotoxins have not been studied by OMICS approaches, as no research articles were found in PubMed for the cyclic imine group, such as spirolides, pinnatoxins, portimines, pteriatoxins, or gymnodimines. Likewise, the different species of dinoflagellates can produce a wide variety of potentially toxic metabolites, which have not been analyzed for their effects by OMICS approaches.

Secondly, most of the OMICS-based studies on marine toxins describe the organism response immediately after an acute exposure. As acute toxin exposure can lead to chronic illness or sequelae, future works based on global approaches should focus on this aspect. Moreover, there is an urgent need to consider the risk of chronic low-level marine toxin exposure. Global approaches such as proteomics are preferable to describe alterations in brain proteins, which could be linked to pathological mechanisms involved in neurodegenerative diseases [48]. 

Third, beyond OMICS approaches, epigenomics has not yet been studied in the field of marine toxin research. Epigenomics studies chemical modifications of the DNA and of the proteins organizing the three-dimensional structure of genomic DNA [138]. By studying changes in DNA methylation and/or histone acetylation, epigenomics will be an interesting approach to better characterize the biological effects of marine toxin chronic exposures. 

Fourth, the use of proteomics in the field of marine toxin research is quite basic and is currently only focused on the characterization of protein abundance changes, rarely using high-throughput technologies. Moreover, a major concern in proteomic studies is the characterization of post-translational modifications (PTM), such as phosphorylation, glycosylation, or acetylation [139]. As marine neurotoxins act on receptors that take part in neuron depolarization or in synaptic transmission, it could be interesting to investigate this aspect in order to understand how exposure to these toxins could modify the regulation of neuronal proteins.

Finally, applying single-OMICS analyses can provide a global view of the response of an organism exposed to a toxin, but only by f ocusing on a type of macromolecule (mRNA, protein, lipid, etc.). They cannot provide a systemic understanding of toxicity pathways or adverse outcome pathways [140]. The next challenge will certainly be the integration of multi-OMICS datasets in order to bring about an improvement in the confidence in detecting the global response/adaptation of an organism to the acute or chronic exposure to distinct marine neurotoxins. Indeed, each OMICS data contain specific information that is not present in other data, and multi-OMICS integration can help to provide a more comprehensive overview of the biological response to biotoxin exposure. However, as the different types of OMICS have a large number of heterogeneous biological variables and a low number of biological samples, incorporation of different biological layers of information to predict phenotypic outcomes remains challenging. To this end, a considerable number of computational tools have been developed over the years, with their advantages and limitations [141]. Briefly, these statistical and computational approaches include unsupervised data integration algorithms (matrix factorization methods, Bayesian methods, and network-based methods) and supervised data integration methods (network-based models, multiple kernel learning methods, and multistep analysis based models) [141]. Recently, based on the selection and description of nine matrix factorization approaches dedicated to the integration of multi-OMIC data representative of the state of the art, Cantini et al. [142] developed a tool allowing testing the best approach depending on type of biological goal. 

## Figures and Tables

**Table 1:** Description of articles using a genomic or transcriptomic approach.

Toxins	Species	Consequences	Technique	Article
STX	*Calanus finmarchicus*	Less energy: downregulation of lipid biosynthesis, growth, and reproduction, and of antioxidants enzymes.	RNA-Seq	[10]
	*Mytilus chilensis*	Involvement of immune response with PRRs.	RNA-Seq and RT-qPCR	[11]
	*Mytilus chilensis*	SOD and CAT as early potential biomarkers of pollution/involvement of immune response.	qPCR	[12]
	*Mytilus galloprovincialis*	Involvement of immune response.	RNA-Seq	[13]
	*Chlamys farreri, Patinopecten yessoensis*	GPx as protectors in the kidneys against the oxidative stress.	RNA-Seq	[14]
	*Chlamys farreri*	Upregulation of SODs in the hepatopancreas and the kidneys.	RNA-Seq	[15]
	*Patinopecten yessoensis, Crassostrea gigas, Lottia gigantean*	Close relation between the expression of SLCs and STX accumulation in the hepatopancreas.	RNA-Seq	[16]
DA	*Danio rerio*	Upregulation of neurodegeneration, especially memory functions.	Microarray and RT-qPCR	[17]
	*Danio rerio*	Five genes of interest involved in glutamate receptor, neurodegeneration, anti-inflammatory, and apoptotic responses.	RT-PCR	[18]
	*Zalophus californianus*	Three dysregulated genes already found in AD studies.	Microarray	[19]
	*Mytilus galloprovincialis*	Upregulation of detoxification processes, the response against the oxidative stress, and immunological processes.	RNA-Seq	[20]
	*Aequipecten opercularis*	Upregulation of detoxification and immunological processes.	RNA-Seq	[21]
	*Pecten maximus*	Upregulation of detoxification and immunological processes.	RNA-Seq	[22]
BTX	*Trichechus manatus latirostris*	Upregulation of the immune and neurodegenerative pathways.	RNA-Seq	[23]
	*Tursiops truncatus*	BTX as haptens that induce an inflammatory immune response.	Restriction site-associated DNA sequencing	[24]
	*Mus musculus* (BALB/C)	No direct binding between BTX6 and AhR.	DNA microarray	[25]
CTX	*Mus musculus* neurons (primary cultures of mice cortical neurons)	Activation of the mu1 opioid related to the hypothermia induced by CTX treatment.	Microarray	[26]
	*Mus musculus* (C57BL6)	Inflammatory response.	Microarray and RT-qPCR	[27]
	*Mus musculus* (C57BL6)	Histamine mediating inflammatory response may cause asthma-like symptoms/dysregulation in detoxification metabolism.	Microarray and RT-qPCR	[28]
	*Mus musculus* (C57BL6)	Detoxification metabolism in the hepatocytes/dysregulation of immune and inflammation systems.	Microarray and RT-qPCR	[29]
	*Homo sapiens*	Inflammatory response, haplotypes *DQ2,* and *DQ8* over-represented.	Microarray and RT-qPCR	[30]
BMAA	*Rattus norvegicus* cells (OEC)	Dysregulations of apoptosis, excitotoxic pathway, aggregation and degradation of proteins, and cell homeostasis/upregulation of VDAC1.	Microarray and RT-qPCR	[31]
TTX	*Octopus bimaculoides*	TTX resistance: hypothesis of NaV domain 1 mutation.	DNA sequencing and RNA-Seq	[32]
	*Takifugu rubripes*	Involvement of the immune system in the liver.	RT-PCR	[33]
	*Takifugu rubripes*	Involvement of the immune system in the liver.	Microarray	[34]
	*Takifugu rubripes*	Modulation of the NaV kinetic.	Microarray and RT-qPCR	[35]
	*Nassarius succinctus, Nassarius variciferus*	Dysregulations of detoxification and immune systems/NaV mutation.	RNA-Seq	[36]
	*Pleurobranchaea maculata*	Difference in TTX levels due to exogenous factors.	PCR amplification and genotyping	[37]

**Table 2:** Description of articles using a proteomic approach.

Toxins	Species	Consequences	Technique	Article
STX	*Mus musculus* cells (N2A)	Dysregulated proteins in accordance with genotoxicity and neurotoxicity induced by STX/downregulation of proteins suggesting membrane depolarization.	2D DIGE and MALDI-TOF-MS	[41]
	*Mus musculus* (C57BL/6NJ)	Close relationship between dysregulated proteins in long-term effects and neurodegenerative diseases.	TMT labeling and LC-MS/MS	[48]
DA	*Zalophus californianus*	ApoE as indicator of chronic DAT.	2D DIGE and LC-MS/MS	[42]
	*Zalophus californianus*	Upregulation of CSF proteins involved in neurodegenerative pathway and antiapoptotic response.	Label-free LC-MS/MS	[50]
BTX	*Oryzias melastigma*	Dysregulation of calcium homeostasis/downregulation of proteins involved in tissue integrity	2D DIGE and MALDI-TOF/TOF	[43]
	*Porites astreoides*	Downregulation of redox homeostasis, energy metabolism, and ROS production.	iTRAQ and LC-MS/MS	[45]
BMAA	*Danio rerio*	Neurocytotoxic effect/dysregulation of endocannabinoid system.	nanoLC-MS/MS	[51]
	*Homo sapiens* tissue (human brain)	Misincorporation of BMAA in de novo synthesis.	LC-MS/MS	[52]
	*Mus musculus* cells (NSC-34)	Link between neurodegenerative diseases and dysregulation of NRF2.	SILAC and nanoLC-MS/MS	[47]
	*Rattus norvegicus* cells (OEC)	Dysregulation of apoptosis, excitotoxic, cell homeostasis pathways/link with proteins involved in AD and ALS.	nanoLC-MS	[31]
	*Mus musculus* cells (NSC-34)	Dysregulation of apoptosis, excitotoxic, cell homeostasis pathways.	LC-MS/MS	[53]
	*Rattus norvegicus* (wistar)	Dose-dependent increase in neuronal pathways and sex-dependent increase in neuropeptides.	nanoLC-MS/MS	[54]
	*Rattus norvegicus* (male wistar)	Memory impairments: decrease in MBP.	MALDI IMS	[55]
	*Rattus norvegicus* (male wistar)	Downstream response to the BMAA-induced intracellular formation of fibrils.	LC-MS/MS	[56]
TTX	*Octopus kaurna, Hapalochlaena maculosa*	Hyaluronidase as an indicator of TTX production.	LC-MS/MS	[57]

**Table 3:** Description of articles using a metabolomic approach.

Toxins	Species	Consequences	Technique	Article
BTX	*Oryzias melastigma*	Dysregulation of metabolites involved in neural excitotoxicity.	LC-MS/MS	[63]
	*Danio rerio*	Dysregulation of excitotoxic, carbohydrate, and energy metabolisms related to BTX mechanism.	HR-MAS-NMR	[64]
BMAA	*Homo sapiens* cells (SH-SY5Y)	Interference of BMAA with fundamental metabolic pathways related to neurotransmission.	NMR and LC-MS	[65]
	*Rattus norvegicus* (male wistar)	Reorganization of metabolic program to increase energy.	NMR	[66]
	*Danio rerio*	Reorganization of metabolic program to increase energy/ROS production and decrease in protection against excitotoxicity and oxidative stress.	HR-MAS-NMR	[68]
	*Rattus norvegicus* (HSD), *Mus musculus* (B6C3F1/N)	Misincorporation into proteins not proven in urine: excretion problem?	LC-MS/MS	[69]
